# Scaling relationships between the total number of leaves and the total leaf area per culm of two dwarf bamboo species

**DOI:** 10.1002/ece3.70002

**Published:** 2024-07-15

**Authors:** Chengkang Wang, Yi Heng, Qingwei Xu, Yajun Zhou, Xuyang Sun, Yuchong Wang, Weihao Yao, Meng Lian, Qiying Li, Liuyue Zhang, Ülo Niinemets, Dirk Hölscher, Johan Gielis, Karl J. Niklas, Peijian Shi

**Affiliations:** ^1^ Co‐Innovation Centre for Sustainable Forestry in Southern China, College of Landscape Architecture Nanjing Forestry University Nanjing China; ^2^ Bamboo Research Institute, College of Ecology and Environment Nanjing Forestry University Nanjing China; ^3^ Institute of Agricultural and Environmental Sciences Estonian University of Life Sciences Tartu Estonia; ^4^ Estonian Academy of Sciences Tallinn Estonia; ^5^ Tropical Silviculture and Forest Ecology University of Göttingen Göttingen Germany; ^6^ Department of Biosciences Engineering University of Antwerp Antwerp Belgium; ^7^ School of Integrative Plant Science Cornell University Ithaca New York USA

**Keywords:** coefficient of variation, foliage length‐times‐width equation, landscape plant, Montgomery equation, power‐law function, scaling theory, self‐shading

## Abstract

Total leaf area per plant is an important measure of the photosynthetic capacity of an individual plant that together with plant density drives the canopy leaf area index, that is, the total leaf area per unit ground area. Because the total number of leaves per plant (or per shoot) varies among conspecifics and among mixed species communities, this variation can affect the total leaf area per plant and per canopy but has been little studied. Previous studies have shown a strong linear relationship between the total leaf area per plant (or per shoot) (*A*_T_) and the total number of leaves per plant (or per shoot) (*N*_T_) on a log–log scale for several growth forms. However, little is known whether such a scaling relationship also holds true for bamboos, which are a group of Poaceae plants with great ecological and economic importance in tropical, subtropical, and warm temperate regions. To test whether the scaling relationship holds true in bamboos, two dwarf bamboo species (*Shibataea chinensis* Nakai and *Sasaella kongosanensis* ‘Aureostriatus’) with a limited but large number of leaves per culm were examined. For the two species, the leaves from 480 and 500 culms, respectively, were sampled and *A*_T_ was calculated by summing the areas of individual leaves per culm. Linear regression and correlation analyses reconfirmed that there was a significant log–log linear relationship between *A*_T_ and *N*_T_ for each species. For *S. chinensis*, the exponent of the *A*_T_ versus *N*_T_ scaling relationship was greater than unity, whereas that of *S. kongosanensis* ‘Aureostriatus’ was smaller than unity. The coefficient of variation in individual leaf area increased with increasing *N*_T_ for each species. The data reconfirm that there is a strong positive power‐law relationship between *A*_T_ and *N*_T_ for each of the two species, which may reflect adaptations of plants in response to intra‐ and inter‐specific competition for light.

## Graphical abstract

There is a significant correlation between the total leaf area (TLA) and the total number of leaves per plant (NL). Self‐shading influences the TLA versus NL scaling relationship.

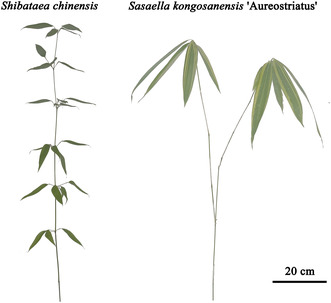

## 1 INTRODUCTION

The lamina area of leaves and its scaling with respect to lamina dry mass can reflect the efficiency of light interception and life‐history strategies (Poorter et al., 2009; Westoby et al., 2002; Wright et al., 2004). However, the above‐ground architectural structure of plants and its influence on the number and distribution of leaves are seldom studied directly, although it is usually influenced by the branching patterns of adjacent plants (Küppers, 1989; Sumida et al., 2002). Total leaf area per plant (*A*_T_) can reflect the photosynthetic capacity of plants, but in practice, it is time‐consuming or not feasible to non‐destructively measure *A*_T_ especially in trees with extensive canopies. In plant ecology, canopy leaf area index (LAI), total leaf area per unit ground area, is widely used to describe canopy structure and to quantify whole canopy photosynthetic potential (Bréda, 2003). LAI at the stand scale can be estimated by destructive harvesting, litter collection, or ground or aerial optical methods (Bréda, 2003). Because attribution of canopy leaf area to individual plant stems is not possible for LAI estimated by litter collection, and is difficult and imprecise for optical methods, *A*_T_ often remains unknown. Optical methods for LAI estimation are particularly problematic for strongly clumped canopies where there is a large overlap among leaves within shoots, branches, and crowns (Niinemets, Al Afas, et al., 2004; Niinemets, Cescatti, & Christian, 2004; Valladares & Niinemets, 2007). Because *A*_T_ is the sum of individual lamina area (*A*) for all individual leaves per plant, the total number of leaves per plant (*N*_T_) can be used to assess *A*_T_ provided average *A* is relatively invariable in the canopy. Given a small variation in *A* among the leaves on a plant, *A*_T_ is expected to proportionally increase with increasing *N*_T_. However, many plant species are known to produce leaves differing in size and shape and to respond adaptively to intra‐ and interspecific competition for light (Sumida et al., 2002). Consequently, a direct proportional (isometric) relationship between *A*_T_ and *N*_T_ is not necessarily the case.

Although scaling relationships have been reported for many organic functional traits (e.g., the leaf mass versus lamina area, the tree height versus the diameter at breast height, insect body mass versus the total wing area, avian egg volume versus surface) (Niklas, 1994; Niklas et al., 2007; Shi et al., 2023; Shi, Jiao, et al., 2022; Sumida et al., 2013), only a few studies (Koyama et al., 2012; Koyama & Smith, 2022) have tested and analyzed the scaling relationship between *A*_T_ and *N*_T_ despite the importance of these two measures of whole plant photosynthetic potential. Koyama et al. (2012) reported a significant linear relationship between *A*_T_ and *N*_T_ on a log–log scale for 208 leaves on the 29 *Cardiocrinum cordatum* rosettes, and the scaling exponent of *A*_T_ and *N*_T_ of the perennial herb was found to be greater than unity. Smith et al. (2017) reported bivariate relationships between the mean leaf area (*A*_M_, i.e., *A*_T_/*N*_T_) versus *N*_T_ at the shoot level for several woody species. This is particularly true for tree species with crowns composed of thousands to millions of leaves, making the direct (and destructive) measurements of *A*_T_ time‐consuming and impractical (Reich et al., 2004). Different problems arise when examining most herbaceous species as a consequence of intraspecific variation in leaf shape and geometry (den Dubbelden & Verburg, 1996; García‐Pérez, 2012). In addition, the comparatively small number of leaves per plant for many herbaceous species precludes robust correlation analyses owing to the small sample sizes. Nevertheless, prior work has reported a negative scaling relationship between leafing intensity, defined as the ratio of the number of leaves per shoot (or per stem) to the shoot (or stem) size, and mean individual leaf mass (Huang et al., 2015; Kleiman & Aarssen, 2007; Scott & Aarssen, 2012; Whitman & Aarssen, 2010; Yan et al., 2012). Unfortunately, these and other studies associated with the leafing intensity theory have not directly explored the scaling relationship between *A*_T_ and *N*_T_. In addition, apart from several studies (Koyama et al., 2012; Koyama & Smith, 2022; Smith et al., 2017), most previous studies used individual leaf dry mass to represent leaf size rather than individual leaf area, presumably because the latter is more tedious to measure, and none examined the scaling relationship between *A*_T_ and *N*_T_, which is hypothesized to correlate with differing degrees of self‐shading.

**FIGURE 1:**
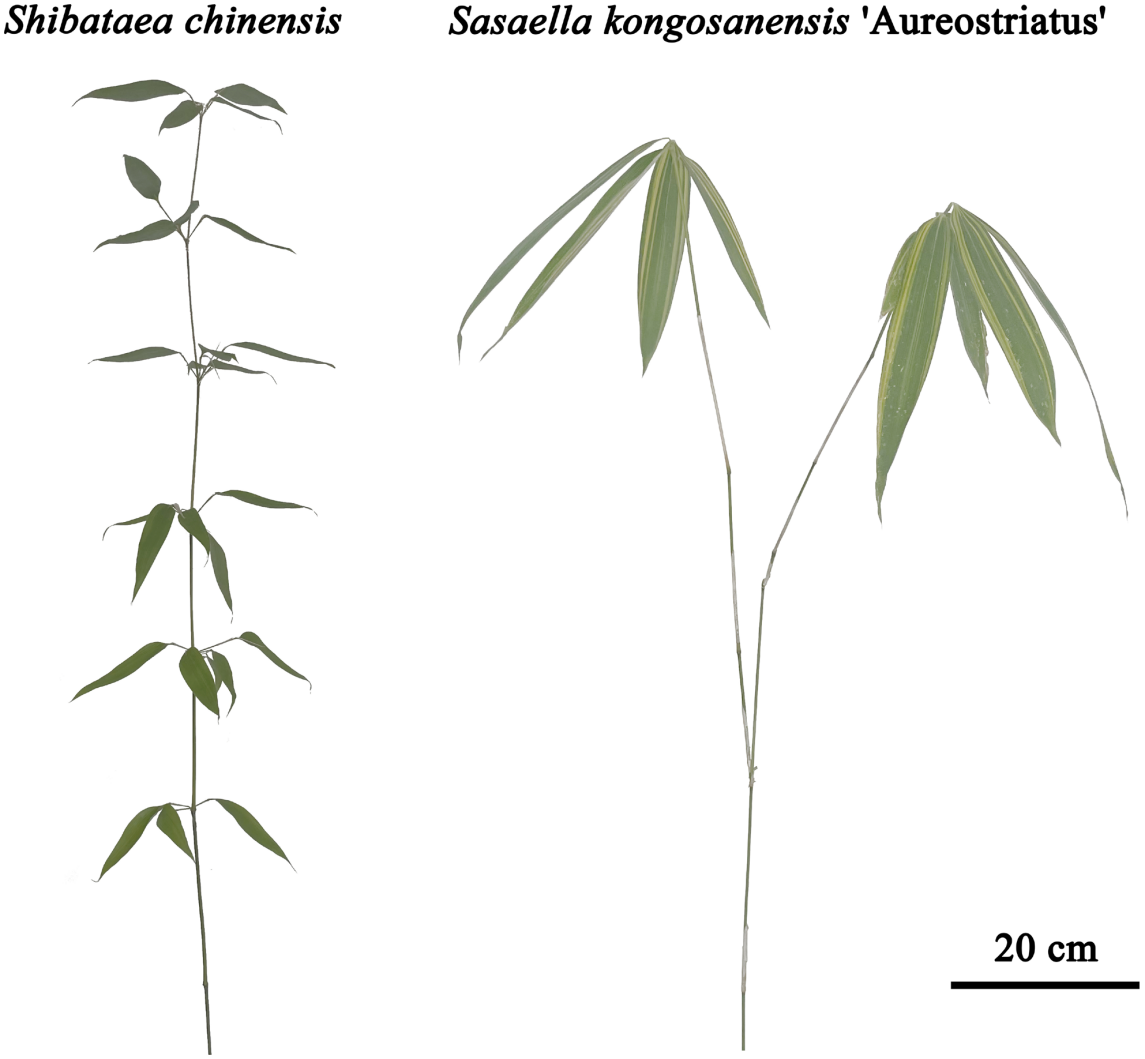
Representative examples and schematics of the culms of *Shibataea chinensis* Nakai (left) and *Sasaella kongosanensis* ‘Aureostriatus’ (right).

In spite of the fact that previous studies have already found a strong scaling relationship between *A*_T_ and *N*_T_ in other plant species (Koyama et al., 2012; Koyama & Smith, 2022; Smith et al., 2017), little is known about whether such a scaling relationship holds true for bamboos, which play very important roles in ecology especially terrestrial carbon uptake and economy. Specifically, we hypothesized that the *A*_T_ versus *N*_T_ scaling relationship will differ among species as a function of self‐shading that in turn depends on shoot architecture (i.e., the manner in which leaves are displayed within the shoot's branching structure) (Figure 1). If correct, the scaling exponent of *A*_T_ versus *N*_T_ for a strongly self‐shaded plant is predicted to be greater than unity (because more leaf area is needed to compensate for shading), whereas the scaling exponent of *A*_T_ versus *N*_T_ for less self‐shaded plant is predicted to be equal to or be smaller than unity (because less leaf area is required to intercept sufficient quantities of light to sustain growth). To test these predictions, we examined two dwarf bamboo species, *Shibataea chinensis* Nakai and *Sasaella kongosanensis* ‘Aureostriatus’, both growing at the same site. We hypothesize that the two types of branching patterns (i.e., the vertical distribution and clumping distribution) can have different effects on the *A*_T_ versus *N*_T_ relationships because of different degrees of leaf self‐shading. The leaves from 480 culms of *S. chinensis* and 500 culms of *S. kongosanensis* ‘Aureostriatus’ were sampled and *A*_T_ was calculated by summing the areas of individual leaves per culm. The *A*_T_ versus *N*_T_ log–log scaling relationships of culms were analyzed. In addition, the coefficient of variation (CV) of leaf area among the individual leaves per culm was calculated to determine the CV versus *N*_T_ scaling relationship to determine whether variation in leaf area increases as a function of increasing number of leaves per culm.

## 2 MATERIALS AND METHODS

### 2.1 Study stands and plants

The study stands of *S. chinensis* (118°48′53″ E, 32°4′52″ N) and *S. kongosanensis* ‘Aureostriatus’ (118°48′58″ E, 32°4′37″ N) are both located in the Nanjing Forestry University Campus, Nanjing, Jiangsu, China. The mean annual temperature is 15.6°C, the mean annual cumulative precipitation is 1058 mm, the mean annual relative humidity is 75.7%, and the mean annual sunshine duration is 2038 h for Nanjing according to the climatic data recorded between 1951 and 2012 (data source: the China Meteorological Data Net; https://data.cma.cn/).

*S. chinensis* and *S. kongosanensis* ‘Aureostriatus’ have been growing on the campus for at least 30 years, and their current distributions reflect different degrees of intraspecific or interspecific competition. The sampled culms of *S. chinensis* were solitarily distributed outside a metal fence of a playground and partially shaded by a neighboring tree (*Cinnamomum camphora* (L.) J. Presl). Field observations and published work indicate that *S. chinensis* can inhibit other species from growing in sites in which it has become established (Pang et al., 2018). Such was the case for the sites sampled for this species in this study. In contrast, *S. kongosanensis* ‘Aureostriatus’ individuals were mixed with *Pleioblastus argenteostriatus* individuals in the sampling site. Because *S. kongosanensis* ‘Aureostriatus’ culms are generally taller than *P. argenteostriatus* culms (Qin et al., 2018) and the leaves of *S. kongosanensis* ‘Aureostriatus’ were aggregated at the top (distal) internodes of a culm (Figure 1), the leaves of *S. kongosanensis* ‘Aureostriatus’ were generally unshaded by the neighboring species. It is worth noting that the phylogenetic placement of *S. kongosanensis* ‘Aureostriatus’ in the genus *Sasaella* Makino has been confirmed recently by studying floral morphology (Lin et al., 2017) and chloroplast genome variation (Zhou et al., 2022). The same plants used in these studies were used in the present study.

These two species were selected for study because (1) their leaves are geometrically similar and sufficiently simple that leaf area can be quantified non‐destructively (as well as empirically) (Lin et al., 2016; Shi et al., 2015, 2018), (2) the vertical distribution patterns of leaves within a culm and the degree of leaf aggregation (clumping) differ significantly, and (3) the culms of both species produce sufficiently large numbers of leaves to yield statistically robust sample sizes (Figure 1). Specifically, *S. kongosanensis* ‘Aureostriatus’ produces sparsely branched and thicker culms bearing larger leaf laminae compared to *S. chinensis* (Figure 1). These differences conform with Corner's rules, which states that species with less ramified shoots will produce larger branches and larger leaves (Corner, 1949; Lauri, 2019). Field observations, indicate that the degree of self‐shading of *S. chinensis* is larger than that of *S. kongosanensis* ‘Aureostriatus’. It therefore becomes feasible to examine and compare whether the scaling exponents of *A*_T_ versus *N*_T_ differed between the two species. The two species presented an additional advantage because both produce a limited but sufficiently large number of leaves per culm, which allowed the measurement of *A*_T_ and *N*_T_ for robust statistical sample sizes.

### 2.2 Leaf data acquisition

For *S. chinensis*, 240 and for *S. kongosanensis* ‘Aureostriatus’, 500 culms were sampled from the stands of each species. The leaves of each culm were harvested, the pseudo‐petioles were removed, and the leaves were individually scanned with an Epson scanner (V550, Epson Indonesia, Batam, Indonesia) at 600 dpi resolution. The resulting images were converted to black and white images and saved as bitmap images at 600 dpi by Adobe Photoshop (version 13.0; Adobe Systems Incorporated, San Jose, CA, USA). The MATLAB (version ≥2009a; MathWorks, Natick, MA, USA) procedure developed by Shi et al. (2018) and Su et al. (2019) was used to extract the planar coordinates of the lamina edges, and the “bilat” function in the “biogeom” package (version 1.3.5; Shi, Gielis, & Quinn, 2022) based on R software (version 4.2.0; R Core Team, 2022) was used to calculate lamina area (*A*), length and width of each of the 740 (240 + 500) culms of collected leaves (Tables S1 and S2).

In addition, to increase the sample size of *S. chinensis*, we harvested another set of 240 culms, and measured the lamina length (*L*) and width (*W*) of each leaf with a ruler (Table S3). For these leaves, *A* was estimated by 2/3*LW*, that is, the Montgomery equation with the proportionality coefficient of 2/3 (Schrader et al., 2021). To compare the results of empirically measured lamina area with the results of estimating lamina area as 2/3*LW*, we sampled 240 individual leaves of *S. chinensis* measured *L* and *W* by a ruler, and determined the *A* by scanning leaf images (Table S4). *A* of each leaf was then also estimated using the Montgomery equation, that is, 2/3*LW*, as recommended by Schrader et al. (2021). The reason that all the leaves of all the additional 240 culms of *S. chinensis* were not scanned was predicated on not biasing sample sizes and prior research, that is, the total number of the leaves per culm (on average, 21 leaves per culm) was larger than that of *S. kongosanensis* ‘Aureostriatus’ (on average, 4 leaves per culm), and the prediction accuracy of the Montgomery equation in estimating the lamina area of bamboo leaves has been validated previously (Lin et al., 2016; Shi et al., 2021).

### 2.3 Data analysis

To test the validity of the Montgomery equation, we used regression protocols to determine whether the empirically measured *A* versus estimated *A* scaling relationship was isometric. The Montgomery equation assumes a proportional relationship between *A* and the product of *L* and *W*, that is, A=kLW, where *k* is the proportionality coefficient (theoretically predicted to numerically equal 2/3). When the two sides of the Montgomery equation are both log‐transformed to stabilize the variance of *A*, the Montgomery equation takes the form $\log\left(A\right)=a+\log\left(\mathrm{LW}\right)$, where *a* = log(*k*). Linear regression protocols were used to determine the 95% confidence intervals (95% CIs) of *a* and to test whether the estimate of the proportionality coefficient in the Montgomery equation is (approximately) equal to 2/3, as suggested by Schrader et al. (2021).

To test whether the scaling of total leaf area per culm (*A*_T_) versus the total number of leaves per culm (*N*_T_) follows a power–law relationship, we used the following equation (Niklas, 1994) to fit the log‐transformed data:

y=γ+αx,

where *y* = log(*A*_T_), *x* = log(*N*_T_), γ is the natural log transformation of the normalized constant, and α is the scaling exponent of *A*_T_ versus *N*_T_. As *N*_T_ can be precisely determined, ordinary least‐squares regression protocols were used to determine the numerical values of γ and α. The coefficient of variation (CV) of *A* among the individual leaves per culm was calculated as:

$$\mathrm{CV}=\frac{\mathrm{SE}}{A_{M}}\times 100\%,$$

where SE and *A*_M_ represent the standard error of leaf areas and mean leaf area per culm. The strength of the correlation between CV and *N*_T_ was assessed by the Pearson correlation coefficient. We also tested whether *A*_M_ tends to decrease as *N*_T_ increases using ordinary least‐squares regression protocols. In addition, we tested the significance of the differences in the total leaf area, mean leaf area, mean ratio of leaf width to length, and total number of leaves per culm between the two species using the *t*‐test after log‐transforming these values to reduce the skewness of the distributions. All statistical analyses were carried out using the statistical software R (version 4.2.0; R Core Team, 2022) and deemed significant at *p* < .05.

## 3 RESULTS

An important consideration in evaluating the results is the extent to which the vertical distribution of leaves affects the degree of self‐shading per culm. Inspection of the two species examined in this study shows that self‐shading may be higher in *S. chinensis* compared to *S. kongosanensis* ‘Aureostriatus’. The degree of self‐shading becomes important because the mean leaf area per culm (*A*_M_ = *A*_T_/*N*_T_) tends to have different trends between the two species as *N*_T_ increases.

With this concern in mind, the individual leaf lamina area (*A*) of *S. chinensis* ranged from 1.1 to 33.4 cm^2^ (mean ± SE = 10.0 ± 3.3 cm^2^, *n* = 10,106), whereas the *A* of *S. kongosanensis* ‘Aureostriatus’ ranged from 1.3 to 142.3 cm^2^ (mean ± SE = 49.3 ± 15.3 cm^2^, *n* = 2111; means are significantly different according to the *t*‐test: *t* = −117.14; *df* = 2152; *p* < .001).

**FIGURE 2:**
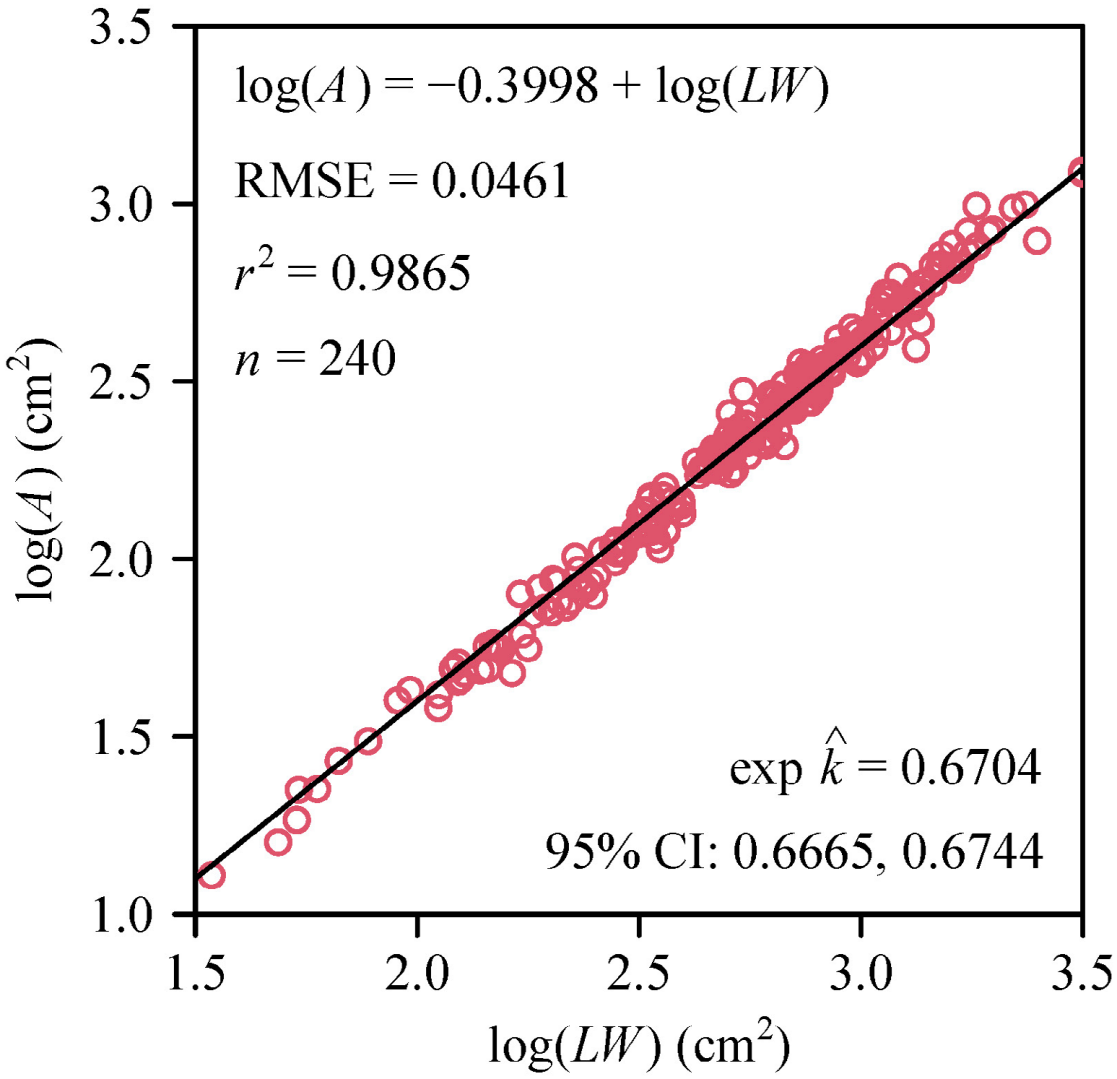
The log–log bivariate relationship between estimates of leaf area using the Montgomery equation based on *LW* and empirically determined leaf area. The small open circles represent the observations of log(*A*) versus log(*LW*); the straight line is the regression line; RMSE is the root‐mean‐square error; *r* ^2^ is the coefficient of determination; *n* is the total number of leaves (the sample size).

For the 240 individual leaves of *S. chinensis*, a statistically robust relationship between empirical and estimated values of *A* (using a proportionality coefficient equal to 2/3) was observed (Figure 2). Specifically, the estimated value of the proportionality coefficient for the relationship between *LW* and *A* was equal to 0.670, approximating closely 2/3. The root‐mean‐square error (RMSE) for the Montgomery equation fit was smaller than 0.05, indicating that the Montgomery equation with a proportionality coefficient of 2/3 could be used to accurately estimate lamina *A*.

**FIGURE 3:**
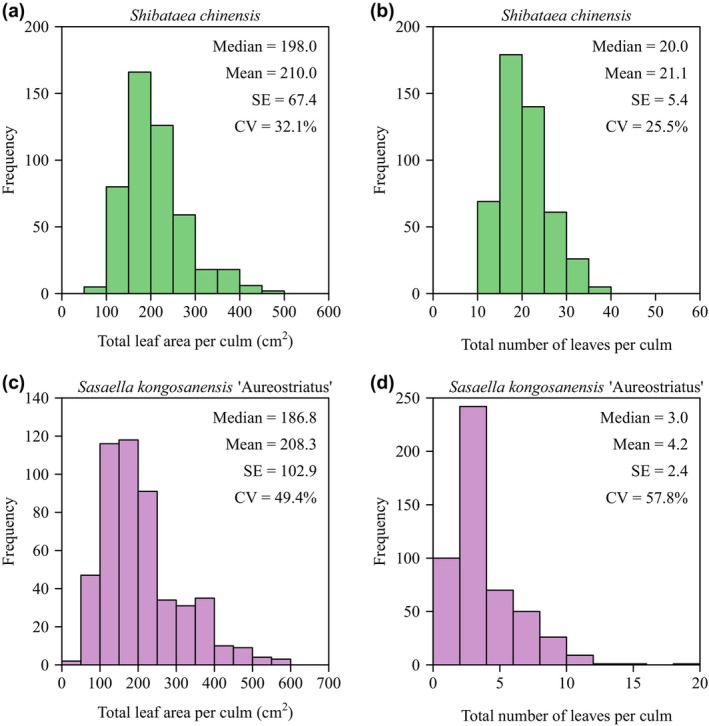
The histograms of total leaf area, and the total number of leaves per culm for *S. chinensis* (a, b) and *S. kongosanensis* ‘Aureostriatus’ (c, d).

**FIGURE 4:**
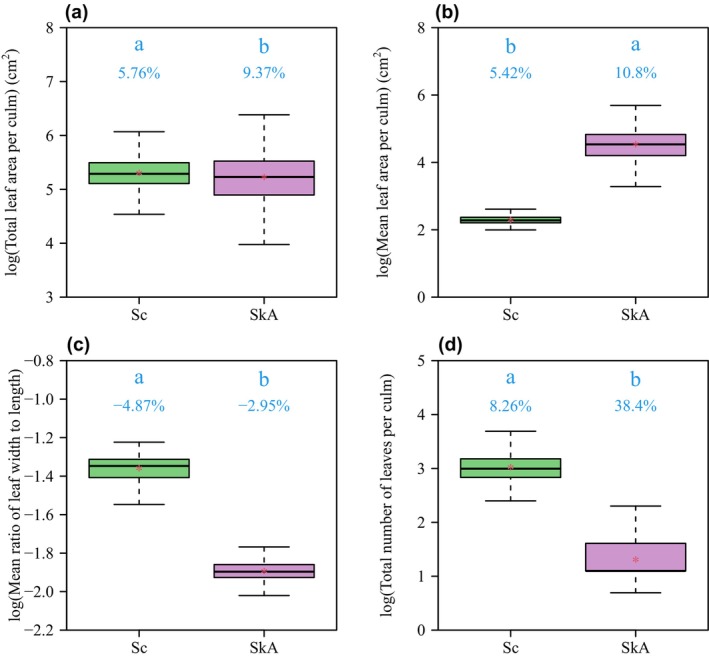
The boxplots of the logarithms of total leaf area (a), mean leaf area (b), mean ratio of leaf width to length (c), and the total number of leaves per culm (d) for the two dwarf bamboo species. The labels of “Sc” and “SkA” on the *x*‐axis represent *S. chinensis* and *S. kongosanensis* ‘Aureostriatus’, respectively. Lowercase letters within each boxplot indicate the significance of mean differences based on the *t*‐test. Different letters represent significant differences (*p* < .05), and the percentages below the letters indicate the coefficients of variation (%). The horizontal solid lines in the boxplot represent the median values, while the red asterisks represent the mean values.

The total leaf area per culm (*A*_T_) of *S. chinensis* was slightly larger than that of *S. kongosanensis* ‘Aureostriatus’ (Figures 3, 4a). However, the mean leaf area per culm (*A*_M_) of *S. chinensis* was significantly smaller than that of *S. kongosanensis* ‘Aureostriatus’ (Figure 4b). *S. chinensis* had more leaves per culm than *S. kongosanensis* ‘Aureostriatus’ (Figures 3 and 4d). The mean ratio of leaf width to length of *S. chinensis* was significantly greater than that of *S. kongosanensis* ‘Aureostriatus’ (Figure 4c). *A*_T_, *A*_M_, and *N*_T_ in *S. kongosanensis* ‘Aureostriatus’ had larger coefficients of variation compared to those in *S. chinensis* (Figure 4).

**FIGURE 5:**
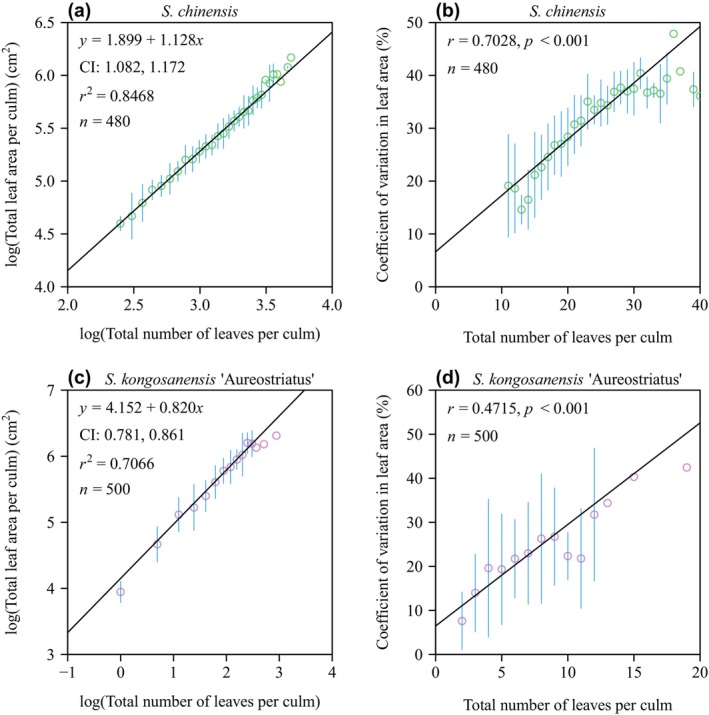
Fitted results for total leaf area per culm versus the total number of leaves per culm plotted on a log–log scale (a, c), and for the coefficient of variation in the individual leaf lamina area among different leaves per culm versus the total number of leaves per culm (b, d). The small red open circles are observations; the vertical blue segments through the small open circles are the standard errors; the straight line is the line regression line. In panels (a) and (c), *y* is the logarithm of the total leaf area per culm; *x* is the logarithm of the total number of leaves per culm; *r* ^2^ is the coefficient of determination; *n* is the sample size, that is, the number of culms used for each species. In panels (b) and (d), *r* is the correlation coefficient; *p* is the *p*‐value of the correlation test; *n* is the number of culms used for each species.

**FIGURE 6:**
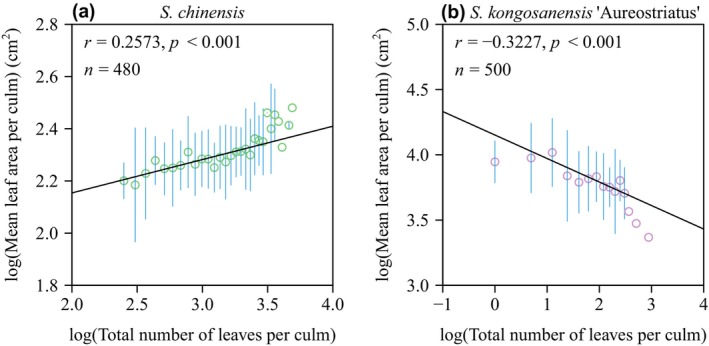
Fitted results for the mean leaf area per culm versus the total number of leaves per culm on a log–log scale for two bamboo specie, *S. chinensis* (a) and *S. kongosanensis* ‘Aureostriatus’ (b). The small red open circles are observations; the vertical blue segments through the small open circles are the standard error; the straight line is the line regression line; *r* is the correlation coefficient; *p* is the *p*‐value of the correlation test; *n* is the number of culms used for each species.

There was a strong log–log linear relationship between *A*_T_ and *N*_T_ for each of the two dwarf bamboo species (*p* < .001; Figure 5). Across all 480 *S. chinensis* culms, the coefficient of determination (*r* ^2^) was .85. The slope of the *A*_T_ versus *N*_T_ scaling relationship had a 95% CI's lower bound value exceeding unity (Figure 5a). Thus, increases in *N*_T_ did not keep pace with increases in *A*_T_. Across all 500 *S. kongosanensis* ‘Aureostriatus’ culms, the coefficient of determination (*r* ^2^) equaled .71. In contrast to *S. chinensis* culms, the slope of the *S. kongosanensis* ‘Aureostriatus’ *A*_T_ versus *N*_T_ scaling relationship had a 95% CI's upper bound value smaller than unity (Figure 5c). For both bamboo species, the CV values of *A* per culm increased with increasing *N*_T_ (Figure 5b,d). However, the mean leaf area per culm (*A*_M_) increased with increasing *N*_T_ for *S. chinensis*, whereas *A*_M_ decreased with increasing *N*_T_ for *S. kongosanensis* ‘Aureostriatus’ (Figure 6).

## 4 DISCUSSION

The data derived from two dwarf bamboo species show that the derivative of the total leaf area per culm (*A*_T_) with respect to the total number of leaves per culm (*N*_T_) can either increase or decrease as a function of *N*_T_ depending on the manner in which leaves are positioned along the length of culms and that the coefficient of variation in the leaf (lamina) area (*A*) among individual‐to‐individual leaves per culm (CV) increases in proportion to *N*_T_. These two features, which likely extend to other species, are discussed in relation to other aspects of light interception.

In the context of the *A*_T_ and *N*_T_ scaling relationship, the lower bound of the 95% CI of the log–log slope of *A*_T_ versus *N*_T_ for *S. chinensis* equals 1.082, and therefore is approximately unity. Thus, *A*_T_ tends to increase isometrically (one‐to‐one) with increasing *N*_T_ for this species. The leaves of *S. chinensis* are distributed more or less evenly along the lengths of culms (see Figure 1), which we attribute to intra‐specific competition for light (Leng & Wang, 2010; Qin et al., 2018), particularly since previous simulations have shown that increases in internodal distances can increase the efficiency of light interception regardless of leaf shape or size, or phyllotactic arrangement (Niklas, 1988; Niklas & Owens, 1989) and because field observations indicate a taller stem, which is achieved by either more of longer internodes and a larger number of leaves improves light interception (Aerts, 1999; Falster & Westoby, 2003). Indeed, *N*_T_ is closely correlated with culm height in *S. chinensis*. There are on average 21 shade‐tolerant leaves per culm. Therefore, a taller culm has more leaves (and therefore more internodes) than a shorter culm. In addition, the mean leaf area of taller culms is larger than that of shorter culms. For an individual culm, the middle and lower internodes of a culm have larger shade‐tolerant leaves with smaller leaf dry mass per unit area (LMA), whereas the upper layers have smaller leaves with larger LMA (Duursma & Falster, 2016; Poorter et al., 2009). This phenomenology also explains why larger *N*_T_ tends to result in a larger variation in *A* because a larger number of leaves is associated with an increase in the size (and shade‐tolerance) of leaves per culm.

In contrast, the leaves of *S. kongosanensis* ‘Aureostriatus’ are not distributed along the entire length of the culm, but rather aggregated at the top (distal) internodes of a culm (see Figure 1). In addition, there is less variation in *A* among leaves compared to *S. chinensis*, perhaps because the leaves are produced and develop approximately at the same time in comparison to the leaves of *S. chinensis*, which develop sequentially as internodes are produced and elongate in a serial‐like manner. This conjecture is supported by the fact that the CV of leaf *A* for *S. kongosanensis* ‘Aureostriatus’ is demonstrably smaller than that of *S. chinensis* (Figure 5b,d). In addition, a positive and a negative log–log scaling relations are observed between mean leaf area per culm (*A*_M_) and *N*_T_ for the two species (Figure 6). However, it merits further investigation on the relationship between the *A*_M_ and leafing intensity (i.e., the ratio of *N*_T_ to the non‐leaf above‐ground biomass) in future investigation. For example, using 24 common deciduous broad‐leaved trees in North America, Kleiman and Aarssen (2007) report that the mean leaf size per shoot has a negative log–log relationship with mean leafing intensity (i.e., the total number of leaves produced by newly emerging shoots, divided by the total volume of shoots). Likewise, Shi et al. (2015) report that *A*_T_ has a negative log–log correlation with the spatial density (i.e., the number of culms per unit ground area) for four species of the dwarf bamboo genus *Indocalamus*. Whether *N*_T_ has a negative correlation with the population density merits further investigation.

**FIGURE 7:**
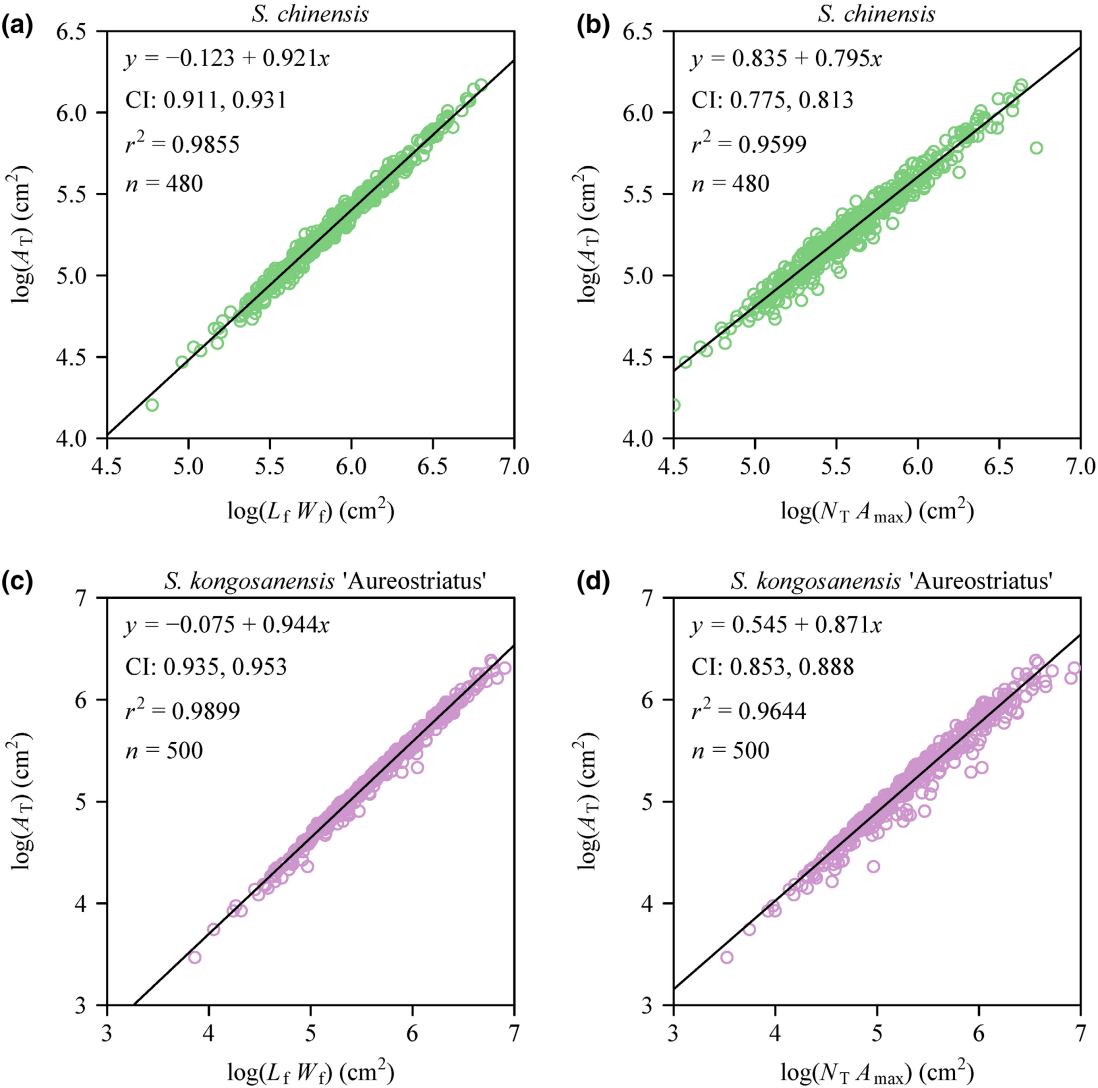
Fitted results for the total leaf area per culm (*A*_T_) versus the product of foliage length (*L*_f_) and foliage width (*W*_f_) per culm plotted on a log–log scale (a,c), and for the total leaf area per culm (*A*_T_) versus the product of the total number of the leaves (*N*_T_) and maximum individual leaf area (*A*_max_) per culm plotted on a log–log scale (b,d). In each panel, CI represents the 95% confidence interval of the slope; the small open circles are observations; the straight line is the line regression line. *r* ^2^ is the coefficient of determination; *n* is the sample size, that is, the number of culms used for each species. Here, *L*_f_ represents the sum of all individual leaf width values per culm; *W*_f_ represents the maximum individual leaf length per culm; *A*_max_ represents the maximum individual leaf area per culm.

Koyama and Smith (2022) proposed a novel model hypothesizing a proportional relationship between *A*_T_ and the product of *L*_f_ and *W*_f_ at the individual plant/shoot level based on the Montgomery equation at the individual leaf level (see Koyama et al., 2012; Schrader et al., 2021; Shi et al., 2019 and references therein). Here, *L*_f_ is referred to as foliage length, representing the sum of all individual leaf width values per plant (or per shoot), and *W*_f_ is referred to as foliage width, representing the maximum individual leaf length per plant (or per shoot). This model, which is called the foliage length‐times‐width equation, actually includes two hypotheses: (i) all leaves per plant (or per shoot) form the analog of “a pinnately compound leaf” and (ii) the analogous pinnately compound leaves across different individual plants of the same plant species are affine in geometry regardless of the morphological variations in the other parts across individual plants (or per shoot). The two hypotheses are apparently reasonable given that the leaf‐shape variation across the individual leaves per plant (or per shoot) is small and the leaf‐size distributions across individual plants exhibit similarity to a great degree for many broad‐leaved plants (Huang et al., 2023; Lian et al., 2023; Shi et al., 2021, 2024). Based on the foliage length‐times‐width equation and other assumptions, especially a hypothesis that individual leaf width (*W*) is proportional to individual leaf length (*L*), Koyama and Smith (2022) obtained two important propositions: $A_{T}\propto N_{T}A_{\max}$ and $A_{T}\propto N_{T}^{\delta }$, where *A*_max_ represents the maximum individual leaf area per plant, and the numerical value of parameter δ is derived to be greater than unity. In addition, they also derived that $A_{M}=A_{T}/N_{T}\propto N_{T}^{\delta -1}$, that is, *A*_M_ is proportional to the δ − 1 power of *N*_T_. Because δ > 1, *A*_M_ is predicted to be an increasing function of *N*_T_. In the present study, the analyses on the *A*_T_ versus *N*_T_ and *A*_M_ versus *N*_T_ scaling relationships of *S. chinensis* (Figures 5a and 6a) conform to the theoretical derivations of Koyama and Smith (2022); however, the estimated scaling exponent of *A*_T_ versus *N*_T_ of *S. kongosanensis* ‘Aureostriatus’ is smaller than unity (Figure 5c), and *A*_M_ is a decreasing function of *N*_T_ (Figure 6b). The converse empirical results for *S. kongosanensis* ‘Aureostriatus’ to the predictions of Koyama and Smith (2022) are likely attributable to a large coefficient of variation (CV) in *N*_T_ (Figure 4d). In addition, we used ordinary least‐squares (OLS) regression to fit the *A*_T_ versus *L*_f_ *W*_f_ and *A*_T_ versus *N*_T_ *A*_max_ scaling relationships for the two bamboo species, and found the slopes on a log–log scale were significantly smaller than unity (Figure 7). The estimated scaling exponents of *A*_T_ versus *L*_f_ *W*_f_ were equal to 0.921 and 0.944 for *S. chinensis* and *S. kongosanensis* ‘Aureostriatus’, respectively (Figure 7a,c), which are reconfirmation of the previous study by Koyama and Smith (2022). In addition, the estimated scaling exponents of *A*_T_ versus *N*_T_ *A*_max_ were equal to 0.795 and 0.871 for *S. chinensis* and *S. kongosanensis* ‘Aureostriatus’, respectively, which largely deviated from the unity (Figure 7b,d). The above <1 slopes were first observed by Koyama and Smith (2022) in five other species. The deviations of the two scaling exponents from unity, as predicted by Koyama and Smith (2022), might result from the hypothesis that *W* is proportional to *L* in their derivation process. In the spite of the fact that *W* is significantly positively correlated with *L*, the 2 one‐dimensional leaf measures tend to have an allometric relationship, and the variation in the *W/L* ratio is found to seriously influence the prediction accuracy of individual leaf area (*A*) based on the hypothesis of $A\propto L^{2}$ (Shi et al., 2019, 2021; Yu et al., 2020). In addition, the variations in the skewness of size distributions and total number of leaves across individual plants (or individual shoots) also tend to influence the *A*_T_ versus *L*_f_ *W*_f_ scaling relationship. Nevertheless, we argue that the foliage length‐times‐width equation and its derivations are still valuable in estimating *A*_T_, which is time‐consuming to directly measure in practice. In addition, whether the foliage length‐times‐width equation and its derivations are applicable to other species merits further investigation.

## 5 CONCLUSIONS

The total leaf area per culm (*A*_T_) was reconfirmed to be a power‐law function of the total number of leaves per culm (*N*_T_) for each of *S. chinensis* and *S. kongosanensis* ‘Aureostriatus’ based on two groups of large sample data sets. The numerical value of the scaling exponent governing the *S. chinensis A*_T_ versus *N*_T_ scaling relationship (i.e., 1.128) exceeded unity and was greater than that governing the *S. kongosanensis* ‘Aureostriatus’ *A*_T_ versus *N*_T_ scaling relationship (i.e., 0.820). The data indicate that increases in *N*_T_ produce disproportionate increases *A*_T_ for *S. chinensis* but the opposite effect is observed for *S. kongosanensis* ‘Aureostriatus’. This difference is attributed to the clustering of leaves on the culms of *S. kongosanensis* ‘Aureostriatus’ and its effect on self‐shading. The mean leaf area per culm increases with increasing *N*_T_ for *S. chinensis*, whereas it decreases with increasing *N*_T_ for *S. kongosanensis* ‘Aureostriatus’. However, the coefficient of variation (CV) in leaf area among different individual leaves per culm increases with increasing *N*_T_ for both bamboo species. Despite the statistically robust scaling relationships observed in our study, it is clear that additional species with different branching patterns and leaf lamina morphologies (particularly those manifesting as well as violating Corner's rules) should be examined both under field conditions and experimentally manipulated light conditions.

## AUTHOR CONTRIBUTIONS

**Chengkang Wang:** Formal analysis (equal); writing – original draft (equal). **Yi Heng:** Investigation (equal); writing – review and editing (equal). **Qingwei Xu:** Investigation (equal); writing – review and editing (equal). **Yajun Zhou:** Investigation (equal); writing – review and editing (equal). **Xuyang Sun:** Investigation (equal); writing – review and editing (equal). **Yuchong Wang:** Investigation (equal); writing – review and editing (equal). **Weihao Yao:** Investigation (equal); writing – review and editing (equal). **Meng Lian:** Investigation (equal); writing – review and editing (equal). **Qiying Li:** Investigation (equal); writing – review and editing (equal). **Liuyue Zhang:** Investigation (equal); writing – review and editing (equal). **Ülo Niinemets:** Writing – review and editing (equal). **Dirk Hölscher:** Writing – review and editing (equal). **Johan Gielis:** Writing – review and editing (equal). **Karl J. Niklas:** Formal analysis (equal); supervision (equal); writing – review and editing (lead). **Peijian Shi:** Formal analysis (equal); supervision (equal); writing – original draft (equal).

## FUNDING INFORMATION

This research received no external funding.

## CONFLICT OF INTEREST STATEMENT

The authors declare that the research was conducted in the absence of any commercial or financial relationships that could be construed as a potential conflict of interest.

## Supporting information


Tables S1–S4

## Data Availability

The datasets for this study are accessible in the Tables S1−S4.
